# MiR-181b: new perspective to evaluate disease progression in chronic lymphocytic leukemia

**DOI:** 10.18632/oncotarget.448

**Published:** 2012-02-18

**Authors:** Rosa Visone, Angelo Veronese, Veronica Balatti, Carlo M. Croce

**Affiliations:** ^1^ Department of Oncology and Experimental Medicine, “G. d'Annunzio” University and Unit of Molecular Pathology and Genomics, Aging Research Center (CeSI), “G. d'Annunzio” University Foundation, Chieti, Italy; ^2^ Department of Molecular Virology, Immunology, and Medical Genetics and Comprehensive Cancer Center, The Ohio State University, Columbus, OH, USA

**Keywords:** miR-181b, diagnosis, CLL

## Abstract

Over the past decades numerous markers of the tumor burden have been discovered in chronic lymphocytic leukemia (CLL). Among these, the microRNAs seem to have a promising role. The development and validation of miRNAs as biomarkers should have significant impact in improving early cancer detection and diagnosis, enhancing therapeutic success, and increasing the life expectancy of patients. We identified miR-181b as a biomarker for the progression of this disease from indolent to aggressive. For this study we used sequential samples from patients with either progressive or stable course of the illness. Here, we discuss more extensively this issue by adding novel findings and introducing a novel approach for monitoring CLL patients.

## INTRODUCTION

B-Chronic lymphocytic leukemia (CLL) is a monoclonal disorder characterized by a progressive accumulation of functionally incompetent B lymphocytes expressing on their surface CD5 and CD19 antigens. Based on cases diagnosed in 2004-2008 from 17 SEER geographic areas, the age-adjusted incidence rate was 4.2 per 100,000 men and women per year with the median age at diagnosis of 72 years (http://seer.cancer.gov/). The clinical course of CLL is highly variable and approximately one third of patients never requires treatment and die, from causes unrelated to CLL; in another third an initial indolent phase is followed by progression of the disease; and the remaining third has aggressive disease at the outset and needs immediate treatment. The traditional staging systems for CLL, Rai in the United States and Binet in Europe, are very good at identifying whom to treat at the time of diagnosis but not at identifying who will later need treatment [1]. Clinical trials have shown that treating early-stage CLL with chlorambucil offers no benefit over delaying treatment until the disease progresses [2]. Albeit the results of this study there is consensus that most patients stage I or II disease that is progressive, should be considered for early treatment [3]. On these basis, over the past few year a number of monotherapies have been explored as the frontline therapy of patients with CLL, without a robust improvement in terms of Progression-Free-Survival (PFS) [4-8]. However, the development of more active therapies could rekindle interest in studying the benefit of early treatment for selected patients with CLL [9, 10]. New trials are ongoing to validate whether patients at the early stage can benefit of early intervention (clinicaltrial.gov).In this context the discovery of biomarker of disease progression could be extremely useful to manage patients at the early stage.

To date numerous biological markers of tumor burden and predictive have been identified (some of them reviewed in [11]) but only few are currently used in the standard management of CLL patients. These include IGHV mutational status, ZAP-70 and CD38 expression levels on gated CD19/CD5 cells, FISH analysis. All of those are markers at the diagnosis: IGVH and ZAP-70 are stable while controversy has developed regarding whether CD38 expression can vary over the course of disease [12]. FISH analysis in the current clinical practice is performed only on new diagnosed patients and can vary or not over time, albeit increased abnormal genetic complexity is associated with advanced stages.

### State-of- the-Art on microRNA as potential biomarkers in CLL

Over the past few years a new class of small genes, miRNAs, have demonstrated to be potential biomarkers of cancer. Increasing experimental evidences have supported the idea of aberrant miRNA expression is involved in cancer pathogenesis [13]. In this section we will give an overview on the role of miRNAs as diagnostic and prognostic tools focusing on chronic lymphocytic leukemia.

Ever since the first association of miRNAs with cancer by Calin et. al [14], it was clear that these genes could play an important role in the clinical management of cancer patients. In this study the down-regulation of the expression of the miR-15a/16-1 cluster located at the chromosome region 13q14, which is frequently deleted in B cell chronic lymphocytic leukemia, was correlated with 13q14 deletion as sole abnormality, thereby with a good prognosis. Subsequently, microarray technologies served as powerful tools to analyze the expression of hundreds of miRNAs and to recognize microRNAs CLL-related. A signature of 25 miRNAs was identified to discriminate CLL cells versus CD5+ normal B cells [15]. MiRNAs could also be seen as prognostic indicators in CLL [16]. By correlating the conventional prognostic markers (IGHV mutation status and/or ZAP-70 expression) with global miRNAs expression were identified 13 genes differentially expressed between CLL cells expressing either unmutated IGHV/ ZAP70+ or mutated IGHV/ZAP70-. This signature included miR- 15a, miR-195, miR-221, miR-23b, miR-155, miR-223, miR29a-2, miR- 24-1, miR-29b-2, miR-146, miR-16-1, miR-16-2, and miR-29c. Six of those were also predicting the time-to-treatment, establishing so the first microRNA signature associated with prognosis in CLL. Subsequently other studies partially confirmed those data, probably due to the different approach or a different control used to identify miRNAs differentially expressed [17] [18]. Stamatopoulos et al widen the investigation by considering the prognostic relevance of miR-29c and miR-223 related to multiple parameters (Binet staging, soluble CD23, β2-microglobulin, lymphocyte doubling time, IGVH status, ZAP70, LPL and cytogenetic abnormalities). Two consequently studies by the same authors showed that the low expression of miR-29c and the miR-223 is associated with the progression from Binet stage A to C; both miRNAs could predict treatment-free survival but only miR-29c can significantly predict the overall survival [19, 20]. This microRNA was also described to have a functional role in indolent CLL [21].

Focusing on another prognostic parameter, the cytogenetics, we conducted miRNA analysis in a cohort of 61 patients with CLL cells carrying several distinct karyotypes (trisomy 12, 13q deletion, 11q deletion, 17p deletion and normal karyotype) [22]. We identified 32 microRNAs able to discriminate the 11q deletion, 17p deletion, trisomy 12, 13q deletion, and normal karyotype cytogenetic subgroups. MiRNAs were also able to stratify aggressive from indolent form of the disease among patients with CLL cells harboring the 17p deletion (miR-181 family, miR-223, and miR-29b/c). With the same intent, Rossi et al analyzed miRNAs expression in CLL cases with chromosome 17p deletion and CLLs with normal 17p and normal karyotype. They found miR-21 expression levels predicting the overall survival and miR-181b expression levels predicting treatment-free survival [23]. Recently Moussay et al. looked at the microRNA expression profile in the plasma of CLL patient rather than CLL cells [24]. They found that certain extracellular circulating miRNAs in patients with CLL are present at levels significantly different compared to healthy donors and that some of them are differentially expressed between ZAP70+ and ZAP70- expressing CLL cells. The combined plasma level of miR-29a, miR-483-5p, miR-195, miR-185, miR-135a* and miR-15a could stratifies ZAP70+ from ZAP70- expressing CLL cells. They also showed that miR-20a in plasma correlates with the severity of the disease.

A different approach finalized to identify predictive markers of the drug resistance in CLL was conducted by Ferracin et al. who analyzed the expression of miRNAs in CLL cells from 17 patients receiving fludarabine mono-therapy. The cells were taken from patients before and 5 days after fludarabine treatment [25]. By whole genome expression analysis they confirmed the defect of P53 pathway in the fludarabine-refractory CLL, previously published by Zenz et al [26], although contrarily to that study, they did not find a significant differential expression of miR-34a between responder and refractory cases to fludarabine treatment in their cohort. Instead miR-21, miR-222 and miR-148a expression were able to predict response to therapy with an accuracy of 80% in the training set and 100% in an independent validation set.

All these miRNAs are proposed as biomarkers in this disease and have been identified by comparing groups of patients classified on the basis of other well-established prognostic markers. However, we believe that a retrospective study in which the patients have been studied in a time frame during which their illness developed a different outcome, could provide important information in discovering the markers for the progression of CLL.

### Mir-181b expression for monitoring the clinical progression of chronic lymphocytic leukemia

In the attempt to search for reliable markers of the progression of this disorder we compared the microRNA profile expression of sequential PBMC samples from 23 patients with a progressive disease [22]. The two sequential samples were chosen so that the last time point is a more aggressive form as compared to its previous counterpart (parameters defined according to Hallek *et al*.[27]). By array technologies we identified several miRNAs differentially expressed between coupled samples.

**Figure 1:**
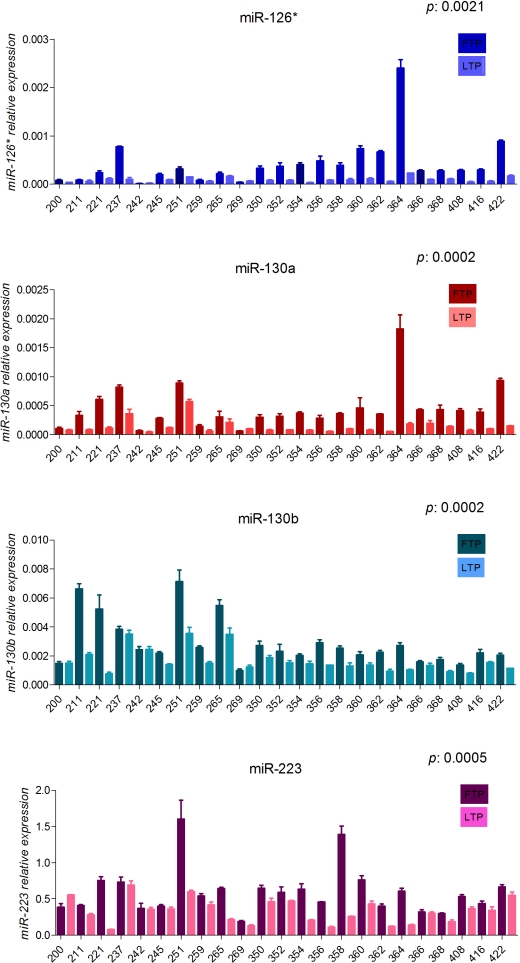
Expression values of miR-126*, miR-130a, miR-130b and miR-223 in sequential samples from patients with progressive disease(training set) Relative expression of the mature miRNAs, in the first time point (dark blocks) and last time point (clear blocks) from sequential samples of CLL patients with progressive disease. The expression has been determined by stem-loop qRT-PCR. Each sample data was normalized to the endogenous reference *RNU44* by using 2^−Δct^ method. *P value* is the result of the paired ttest

**Figure 2:**
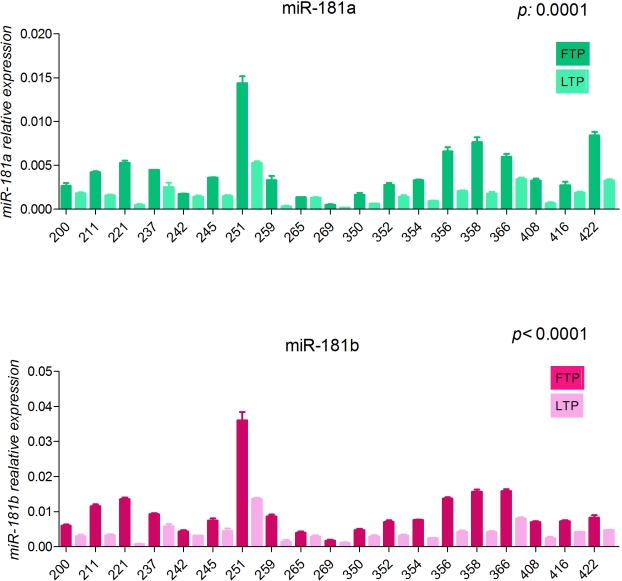
Expression values of miR-181a and miR-181b in sequential samples from patients with progressive disease(training set) Relative expression of the mature miRNAs, in the first time point (dark blocks) and last time point (clear blocks) from sequential samples of CLL patients with progressive disease. The expression has been determined by stem-loop qRT-PCR. Each sample data was normalized to the endogenous reference *RNU44* by using 2^−Δct^ method. *P value* is the result of the paired ttest

Then our study was focused on the most significant differentially expressed, miR-181b, although other interesting miRNAs were also found. Here, we show the technical validation by qRT-PCR of miR-126*, miR-223, miR-130a and miR-130b (Fig. 1). The expression of miR-181a was also analyzed on the same set of samples because it is in the same cluster with miR-181b on chromosome 1 (miR-181a-1 and miR-181b-1) and chromosome 9 (miR-181a-2 and miR-181b-2). MiR-181a shows a similar course as compared to miR-181b (Fig. 2) albeit it did not appear in our analysis because of its very low expression, thereby excluded by the data analysis. We observed that all these miRNAs significantly decrease during the progression of CLL; however only miR-181b was further examined on samples from patients with a stable disease and on a validation set of 78 patients including patients with either progressive or stable disease [28].

We found that miR-181b expression value decreased over the progression of the disease while its expression remained unchanged in patients with a stable course of the disease, all the patients were observed for a comparable time. We validated this result in an independent cohort of samples in which the criteria for defining the form of the disease matched those of the training set with the only exception of the WBC (white blood cell). In this cohort not all the patients with a progressive disease had increasing lymphocytosis. We observed decreased expression of the miR-181b over time also in those cases, suggesting that changes in expression levels of the miR-181b occurred independent of increases in white blood cell counts. Looking closely at the expression value of the miR-181b in sequential samples from a few patients, we observed that not always there is a linear reduction over time. We observed slight fluctuations; thereby we used a decrease of 50% or more in miR-181b levels as the threshold to mitigate potential problems caused by small fluctuations in miR expression in any one sample due to technical or biologic variation. Also, we used the value of ≤ 0.005 at the first time point as cutoff to indicate patients with a progressive status. This value represents the expression level of miR-181b normalized on the RNU44 expression level and was chosen as threshold because it was frequent only in samples from patients with a progressive disease.

These two parameters, decrease of 50% or more between sequential samples and value of ≤ 0.005 at the first time point, were defined as properties and associated with the clinical outcome. Kaplan-Meier curves show that a requirement for treatment is clearly associated with decline of miR-181b expression, thereby patients having the properties more likely experience treatment. Other prognostic markers, such as IGVH mutational status and ZAP-70 expression also stratify patients requiring treatment [29] [30], thus, what is the added value of miR-181b to the current biomarkers?

To answer this question, we analyzed, among the patients with progressive disease included in both training and validation sets, how well the expression of miR-181b could identify patients with progressive/aggressive disease relative to that of other well-established prognostic markers (e.g. CLL-cell expression of unmutated IGHV genes or ZAP-70). Decline of 50% or more between sequential samples and/or value of ≤ 0.005 at the initial observation were noted in serial samples collected from 16 patients that do not have CLL cells with high expression of ZAP-70 and unmutated IGHV, whereas only in 5 patients sequential samples were lacking in properties while CLL cells expressed high ZAP-70 and unmutated IGHV (Table 1). On the other hand, miR-181b was apparently worse predictor of the stable disease compared to the other 2 prognostic markers, in that, among sequential specimens from this patient category, the properties were observed in 7 cases that have CLL cells with low expression of ZAP-70 and mutated IGHV, whereas only 3 cases did not showed the properties and had CLL cells with high expression of ZAP-70 and unmutated IGHV (Table 2). A further analysis of the data shows that the drop of miR-181b that occurs in patients with a stable disease only in one case leads to a lowering of the miR expression level of ≤0.005, threshold that we set as low value in our study for patients with a progressive disease; in all the other cases the expression of miR-181b decreases but stays still high. We did not draw attention to this point in our study because the methodology we used to determine the expression of miR-181b is not absolute, but this is an important consideration that should be taken into account. This allow us to define miR-181b the most significant biomarker of progressive disease at least in the tested cohorts (Table 3 of [28]).

**Table 1:** Molecular features of patients with progressive disease

Patients ID	IGHV Homology(%)	ZAP-70 positive cells (%)	Patients with properties
**200pts**	**≤98**	**≤20**	*
**211pts**	**≤98**	**≤20**	*
**221pts**	≥98	≥20	*
**237pts**	≥98	≥20	
**242pts**	**≤98**	**≤20**	*
**245pts**	≥98	≥20	
**251pts**	≥98	≤20	*
**259pts**	**≤98**	**≤20**	*
**265pts**	≥98	≥20	*
**269pts**	≥98	≤20	*
**408pts**	**≤98**	**≤20**	*
**416pts**	≤98	≤20	
**422pts**	**≤98**	**≤20**	*
**350pts**	**≤98**	**≤20**	*
**352pts**	**≤98**	**≤20**	*
**354pts**	≥98	≥20	*
**356pts**	**≤98**	**≤20**	*
**358pts**	≤98	≥20	*
**360pts**	≤98	≤20	
**362pts**	**≤98**	**≤20**	*
**364pts**	≥98	≥20	*
**366pts**	≥98	≥20	*
**368pts**	≥98	≥20	*
**353pvs**	≥98	≥20	
**363pvs**	≥98	≥20	
**374pvs**	**≤98**	**≤20**	*
**378pvs**	≥98	≥20	*
**381pvs**	≤98	≥20	*
**384pvs**	**≤98**	**≤20**	*
**387pvs**	≥98	≥20	*
**391pvs**	≥98	≥20	*
**396pvs**	≥98	≥20	*
**400pvs**	≥98	≤20	*
**407pvs**	**≤98**	**≤20**	*
**412pvs**	**≤98**	**≤20**	*
**415pvs**	≥98	≥20	*
**418pvs**	≥98	≥20	*
**423pvs**	≥98	≤20	*
**431pvs**	≥98	≥20	*
**436pvs**	≤98	≤20	
**440pvs**	≥98	≥20	*
**483pvs**	≥98	≥20	*
**509pvs**	≤98	≤20	
**523pvs**	≥98	≥20	
**228pvs**	≥98	≥20	*
**234pvs**	≥98	≥20	
**255pvs**	≥98	≤20	*
**262apvs**	**≤98**	**≤20**	*
**329pvs**	≤98	≤20	
**332pvs**	≤98	≤20	
**321pvs**	**≤98**	**≤20**	*
**340pvs**	≥98	≥20	*
**206pvs**	≤98	≥20	*

Blue labels indicate correct prediction of miR-181b expression and discordant prognostic markers IGHV/ZAP-70

**Table 2:** Molecular features of patients with stable disease

Patient ID	IGHV *homology (%)*	*ZAP-70 positive cells (%)*	Patients with properties
**300sts**	≤98	≤20	
**303sts**	≤98	≥20	
**307sts**	≤98	≥20	*
**310sts**	≤98	≤20	
**314sts**	≤98	≤20	
**317sts**	≥98	≤20	*
**323sts**	≤98	≤20	*
**326sts**	≤98	≤20	
**335sts**	≤98	≤20	
**338sts**	≥98	≤20	
**342sts**	≤98	≤20	
**345sts**	≤98	≤20	
**261sts**	≥98	≥20	*
**448svs**	≤98	≥20	
**452svs**	≤98	≤20	
**457svs**	≤98	≤20	
**460svs**	≤98	≤20	
**463svs**	≤98	≤20	
**467svs**	≤98	≥20	
**470svs**	≤98	≥20	
**474svs**	≤98	≤20	
**478svs**	≤98	≤20	
**486svs**	≤98	≤20	
**490svs**	≤98	≤20	
**493svs**	≤98	≤20	
**496svs**	≤98	≤20	
**500svs**	≥98	≤20	
**504svs**	≤98	≤20	
**514svs**	≤98	≤20	
**519svs**	≤98	≤20	*
**529svs**	**≥98**	**≥20**	
**534svs**	≤98	≥20	
**538svs**	**≥98**	**≥20**	
**541svs**	≤98	≤20	
**545svs**	≤98	≤20	*
**550svs**	≤98	≤20	
**554svs**	≤98	≤20	*
**558svs**	≤98	≤20	
**559svs**	≥98	≤20	
**567svs**	≤98	≤20	
**570svs**	≤98	≤20	
**574svs**	≥98	≤20	
**577svs**	≤98	≥20	
**582svs**	≤98	≤20	
**586svs**	≤98	≤20	*
**590svs**	≤98	≤20	
**599svs**	≤98	≥20	
**603svs**	≤98	≥20	
**607svs**	≤98	≤20	
**612svs**	≤98	≥20	
**616svs**	≤98	≤20	
**620svs**	≤98	≤20	
**624svs**	≤98	≤20	

Blue labels indicate correct prediction of miR-181b expression and discordant prognostic markers IGHV/ZAP-70C.

Another point that needs to be discussed concerns the expression of miR-181b relative to the purity of the PBMC samples. The majority of samples from patients with CLL used for our study were PBMCs with high content of CD5/CD19 positive cells; a few of them had a fraction of leukemic CD5/CD19 positive cells around 80% at the initial point. In serial samples from those patients miR-181b expression reflects the same trend of sequential specimens with high content of the leukemic clone at the initial observation. This result make the prediction of the progression for this disorder easier, since hospital laboratories do not need to purify the CD5/CD19 positive cells; qRT-PCR to evaluate the expression of miR-181b can be easily performed on the RNA extracted from the total PBMC population.

## CONCLUSION

There has been extensively evidence that miR-181b is down-regulated in chronic lymphocytic leukemia compared to the normal control [18, 28, 31]. In our study we demonstrated that its expression further decreases during the progression of this disease, suggesting its evaluation as an important tool for monitoring the course of CLL. The miR-181b also has an important biological role in CLL, since it targets MCL1, TCL1, BCL2 and AID. It can be considered as one of the genes, such as *NOTCH1, XPO1, MYD88* or *KLHL6*, recently discovered to be important in this disease [32]. *NOTCH1* has been found mutated in almost half of the patients with CLL B-cells carrying the trisomy 12 [33]. Moreover, conversely to IGHV mutational status and ZAP-70 expression, which are markers at the diagnosis, the expression levels of miR-181b can be evaluated in PBMC of patients over time, providing potential means to monitor for patients who imminently might require therapy. Therefore, our findings introduce a novel approach in the standard care of CLL patients that could benefit either patients with increased risk of progression (ZAP70 +, unmutated IGHV status) or with favorable prognosis (ZAP70- and mutated IGHV status).
